# Noncontact Breathing Pattern Monitoring Using a 120 GHz Dual Radar System with Motion Interference Suppression

**DOI:** 10.3390/bios15080486

**Published:** 2025-07-28

**Authors:** Zihan Yang, Yinzhe Liu, Hao Yang, Jing Shi, Anyong Hu, Jun Xu, Xiaodong Zhuge, Jungang Miao

**Affiliations:** 1 School of Electronics and Information Engineering, Beihang University, Beijing 100191, China; yangzihan_buaa@buaa.edu.cn (Z.Y.); liuyzh23@buaa.edu.cn (Y.L.); yanghao_buaa@buaa.edu.cn (H.Y.); zhuge@buaa.edu.cn (X.Z.); jmiaobremen@buaa.edu.cn (J.M.); 2 Emergency Department, State Key Laboratory of Complex Severe and Rare Diseases, Peking Union Medical College Hospital, Chinese Academy of Medical Science and Peking Union Medical College, Beijing 100730, China; 18409499354@163.com (J.S.); xujunfree@126.com (J.X.)

**Keywords:** dual radar, narrow beam antenna, breathing pattern, motion interference, thoracoabdominal asynchrony measurement, apnea detection

## Abstract

Continuous monitoring of respiratory patterns is essential for disease diagnosis and daily health care. Contact medical devices enable reliable respiratory monitoring, but can cause discomfort and are limited in some settings. Radar offers a noncontact respiration measurement method for continuous, real-time, high-precision monitoring. However, it is difficult for a single radar to characterize the coordination of chest and abdominal movements during measured breathing. Moreover, motion interference during prolonged measurements can seriously affect accuracy. This study proposes a dual radar system with customized narrow-beam antennas and signals to measure the chest and abdomen separately, and an adaptive dynamic time warping (DTW) algorithm is used to effectively suppress motion interference. The system is capable of reconstructing respiratory waveforms of the chest and abdomen, and robustly extracting various respiratory parameters via motion interference. Experiments on 35 healthy subjects, 2 patients with chronic obstructive pulmonary disease (COPD), and 1 patient with heart failure showed a high correlation between radar and respiratory belt signals, with correlation coefficients of 0.92 for both the chest and abdomen, a root mean square error of 0.80 bpm for the respiratory rate, and a mean absolute error of 3.4° for the thoracoabdominal phase angle. This system provides a noncontact method for prolonged respiratory monitoring, measurement of chest and abdominal asynchrony and apnea detection, showing promise for applications in respiratory disorder detection and home monitoring.

## 1. Introduction

In recent years, with the aging of the population and the prevalence of COVID-19, three of the top five global age-standardized causes of death have been directly related to respiration: COVID-19, chronic obstructive pulmonary disease (COPD), and other pandemic-related deaths [1]. Monitoring of respiration is critical for the diagnosis and prognosis of respiratory disease, helps health care professionals to assess respiratory status and the effectiveness of rehabilitation training, and has broad clinical value [2]. Respiratory monitoring is not limited to respiratory frequency; feature parameters such as respiratory amplitude, rhythm, and chest–abdominal coordination extracted from waveforms can also be used to characterize breathing patterns and further diagnose abnormal breathing [3]. For instance, patients with diabetic ketoacidosis present with deep and labored Kussmaul’s respiration [4], while patients with COPD present chemical imbalances and neurologic alterations that result in significant changes in various respiratory dynamics characteristics and breathing patterns, including the appearance of thoracoabdominal asynchrony (TAA) [5].

Traditional respiratory monitoring involves measuring respiratory airflow signals through sensors such as oro-nasal masks, nasal cannulas and spirometers. These devices often cause physiological and psychological stress to the patient, alter the patient’s natural breathing pattern, and do not allow for prolonged continuous monitoring [6]. Modern respiratory monitoring devices are mostly contact and non-invasive, such as life monitors based on the impedance method, respiratory belts based on piezoelectric or piezoresistive sensors, wearable capacitive pressure sensors, and polysomnography (PSG) for sleep apnea measurement. These devices enable reliable respiratory monitoring and are popular in clinical practice and daily life. However, contact measurements still face some problems, such as the discomfort of placing electrodes on the body surface or wearing a respiratory belt for an extended period, the restriction of patient mobility due to cables from the devices, and the risk of cross-infection [7].

Various noncontact approaches have been developed to address these issues, such as solutions based on optical cameras [8], laser interferometers [9], infrared thermography [10], LIDAR systems [11], WIFI sensing [12], and radar [13,14]. Among them, camera-based solutions are affected by lighting conditions and risk compromising privacy. Laser interferometer-based measurements require specialized hardware and complex setups with high cost and operational skill requirements. Infrared thermography methods are susceptible to interference from ambient temperature and have high error rates. LIDAR systems have high accuracy; however, they have poor penetration into clothing and high system complexity and cost. WIFI-based breath measurements are prone to signal interference from other electronic devices.

Previous studies have demonstrated the feasibility of using radar for monitoring respiratory movements by measuring body surface displacements [15]. Radar-based solutions are not constrained by lighting conditions and do not compromise privacy. Radar signals are not affected by the ambient temperature and have better immunity to interference. In recent years, advances in semiconductor technology, especially silicon-based processes such as Complementary Metal-Oxide-Semiconductor (CMOS) and Silicon-Germanium (SiGe), have driven the development of integrated radar circuits [16]. The cost of millimeter-wave radar chips continues to fall, and the availability of a large number of commercially available radar platforms has lowered the threshold for designing radar systems. In addition, the radar’s ability to continuously measure displacement changes in the thoracic or abdominal cavity enables real-time respiratory waveform monitoring, which provides a basis for respiratory pattern extraction.

However, there are still some challenges in estimating breathing patterns using radar. First, most existing systems use a single wide-beam radar sensor reflecting the effect of average human torso motion, which is unable to characterize the local motion of the thoracic and abdominal cavities, as well as the synchronization between them, with a high spatial resolution. Clinically, the coordination of thoracic and abdominal movements is often used as an indication of respiratory distress and dyspnea [17], and TAA provides a method for assessing patients’ respiratory status, the measurement of which is essential for estimating breathing patterns [18]. Second, unavoidable motion interferences during prolonged monitoring can seriously affect the measurement of respiratory patterns and cause errors [19,20,21]. As the displacement of the body’s surface caused by respiratory motion is on the order of millimeters, motion artifacts such as random body movement, sneezing and coughing contaminate the respiratory signal, making subsequent signal analysis and processing difficult. Third, many previous studies mainly extracted only the respiratory frequency, thus neglecting the rich information in the respiratory waveform. Furthermore, most of them utilizing infinite impulse response (IIR) low-pass [22] or band-pass [23] filters to obtain the vital sign signals. However, these filters may change the amplitude and phase of respiration [24], affecting the extraction of feature parameters such as respiration amplitude and TAA. Therefore, a suitable means of reconstructing the respiratory waveform based on preserving the original characteristics of the respiratory signal as much as possible is needed.

**Figure 1:**
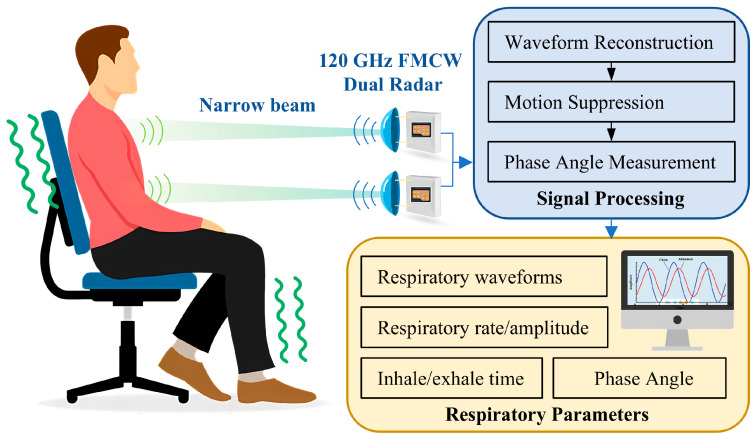
A schematic diagram of the 120 GHz FMCW dual radar system and signal processing flow.

In this work, we propose a novel dual radar system to estimate breathing pattern, as shown in Figure 1. The customized radar sensor is based on a 120 GHz millimeter-wave integrated circuit (MMIC) and inter-radar synchronization is achieved using hardware circuitry. On one hand, the short wavelength of 120 GHz millimeter waves makes it easier to achieve a narrow beam, thus spatially avoiding mutual interference between radars; on the other hand, the system with hardware synchronization circuitry can accurately control the dual radars to be staggered in time to reduce mutual influence. As it is difficult for subjects to remain calm at all times during prolonged measurements, the measured segments of data disturbed by movement contain strong noise, which seriously affects the extracted respiratory parameters. Therefore, a novel method based on adaptive dynamic time warping (DTW) is proposed to detect and exclude the interfered segments, which improves the accuracy of the measurement.

In summary, the contributions of this paper can be highlighted as follows:

(1) This study presents a novel dual radar system for non-contact breathing pattern monitoring using 120 GHz FMCW radars. The system employs customized narrow-beam antennas and temporally staggered radar signals to achieve spatial and temporal separation. This design allows for independent monitoring of chest and abdominal movements, enabling the accurate measurement of thoracoabdominal asynchrony (TAA) and other breathing pattern parameters.

(2) To address the challenge of motion interference, an innovative signal processing method is proposed. Specifically, an adaptive DTW algorithm is adopted, which can automatically distinguish the normal data segment from the interfered segment, resulting in an average signal-to-noise ratio (SNR) improvement of 4.36 dB. The method is also used to identify pathological breathing patterns. Furthermore, the Savitzky–Golay filtering algorithm and the cross-correlation method are utilized to reconstruct the respiratory waveform with the extraction of the chest–abdominal phase angle.

(3) Comprehensive experiments were conducted to validate the efficacy of the proposed system in non-contact breathing pattern monitoring. During experimentation on 35 healthy subjects, 2 patients with chronic obstructive pulmonary disease (COPD), and 1 patient with heart failure in a clinical environment, the system demonstrated a high correlation with reference respiratory belt signals (correlation coefficient: 0.92) and accurate respiratory rate estimation (RMSE: 0.80 bpm). The results also showed a mean absolute error of 3.4° for the thoracoabdominal phase angle, suggesting that the system has potential for clinical applications (<5° [25]).

The rest of this paper is organized as follows. Section 2 describes the dual radar system and the breathing pattern estimation algorithm. Section 3 presents experiments on normal and abnormal breathing patterns. Section 4 evaluates the system performance and discusses future work. Finally, Section 5 concludes the paper.

## 2. Methods

### 2.1. FMCW Radar Principle on Breath Detection

The Frequency-Modulated Continuous Wave (FMCW) radar sensor measures respiration on the principle of the micro-Doppler effect. Specifically, the body surface displacement of the human chest or abdomen modulates the phase of the reflected echo, and the radar detects such small displacement by measuring changes in the phase of the signal. For FMCW quadrature radar systems, the transmitted signal is mixed with the received signal through a mixer to obtain an Intermediate Frequency (IF) signal, which can be approximated as

(1) $$S_{IF}\left(t\right)=A*exp(j(2\pi f_{IF}t+\frac{4\pi x(t)}{\lambda }+\frac{4\pi d_{0}}{\lambda }))$$

where A is the amplitude of the IF signal, $f_{IF}$ is the frequency of the IF signal, λ is the maximum wavelength of the radar carrier, $x\left(t\right)$ is the target motion, and $d_{0}$ is the distance between the human body and the radar antenna.

Compared to continuous wave (CW) Doppler radar, FMCW radar has a certain bandwidth and therefore has a distance resolution, which can be expressed as δ=c/2B, with c being the speed of light, and B being the swept bandwidth. The target distance is proportional to the $f_{IF}$, and therefore the fast Fourier transform (FFT) can be utilized to classify the signal into different range bins. The range bin where the human body is located is selected for processing to reduce the effect of background clutter.

It can be seen from (1) that the change in the phase of the radar IF signal is inversely proportional to the wavelength for a certain motion displacement. Therefore, a higher frequency is good for increasing sensitivity. Millimeter wave radar is a good choice for measuring human vital sign applications. Electromagnetic waves in this band have a sub-millimeter penetration depth into the human body, so radar echoes mainly carry displacement information on the body surface. The detected body displacement $x\left(t\right)$ mainly consists of respiratory and heartbeat components and noise such as body movement, which can be modeled as

(2) $$x\left(t\right)=x_{r}\left(t\right)+x_{h}\left(t\right)+N\left(t\right)$$

where $x_{r}\left(t\right)$ and $x_{h}\left(t\right)$ stand for respiration and heartbeat-induced body surface movements, respectively, and $N\left(t\right)$ represents noise caused by body movement.

The respiratory component is derived from the visually visible rise and fall of the thorax and abdomen caused by contraction and diastole of the diaphragm and intercostal muscles during respiration. Correspondingly, the heartbeat component derives from the body surface displacement and vibration caused by contraction and diastole of the atrial and ventricular muscles as well as the pulse wave traveling through the body. Body movements mainly consist of slow unconscious movements, as well as sudden movements such as coughing and sneezing.

It is important to note that the amplitude of the respiratory motion may exceed half the wavelength, resulting in wrapping of the IF signal phase [26], which needs to be unwrapped. After excluding motion disturbances and heartbeat signals, breathing patterns can be extracted from respiratory waveforms.

### 2.2. Dual Radar Anti-Interference Design

As the radar detects breathing by measuring radial displacements, it should be oriented to the body surface area that undulates the most during respiration to improve accuracy. However, normal human breathing patterns include chest and abdominal breathing [27]. These two breathing patterns produce a large amplitude in the chest or abdomen, respectively, and a small amplitude in other positions. Therefore, the chest and abdomen can be monitored simultaneously by the dual radar to avoid false detection.

**Figure 2:**
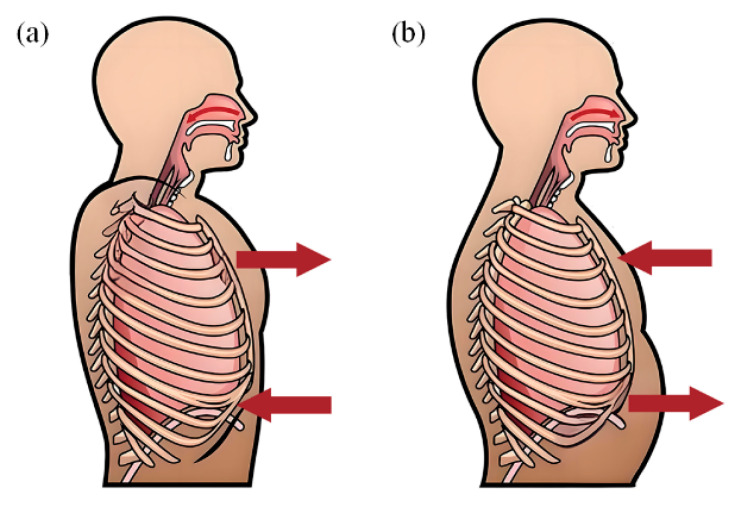
A schematic diagram of paradoxical breathing, the arrows show the direction of chest and abdominal movement. (**a**) During inhalation, the chest expands while the abdominal wall collapses; (**b**) during exhalation, the chest narrows and the abdominal wall expands.

In addition, the asynchrony of chest and abdominal movements is associated with diseases such as chronic obstructive pulmonary disease (COPD) [18], and this asynchrony can be characterized well quantitatively using dual radar. When the patient’s condition is severe with restricted respiratory airflow, it leads to the appearance of paradoxical respiration [28], where the thorax expands while the abdominal wall collapses during inspiration, and the thorax narrows while the abdominal wall expands during expiration, as shown in Figure 2. In order to avoid mutual interference between the radars and accurately measure the phase angle of chest and abdominal motion, the radar antenna and radar signal were carefully designed in this study.

Common radar frequencies for vital signs monitoring include 24 GHz, 60 GHz, 77 GHz, and 120 GHz. Considering the spatial resolution, device size, sensitivity, and penetration required for chest and abdominal respiration measurement applications, the 120 GHz radar frequency was selected.

First, independent chest and abdomen measurements require high spatial resolution. The center distance between the chest and abdomen for adults is about 15–25 cm, so a narrower radar beam can more accurately irradiate the chest and abdomen regions separately, reducing the spatial crosstalk between the chest and abdomen signals and ensuring the physical independence of the radar signals. For children, the chest and abdomen distance is even smaller, so the high resolution also facilitates the system’s adaptability to different populations. The beamwidth of the antenna is proportional to the wavelength. The beamwidth of 60/77 GHz radar is typically greater than 10°; thus, to achieve the same beamwidth as that of 120 GHz radar, the increase in antenna size and weight introduces more limitations to practical applications.

Second, 120 GHz radar has higher sensitivity. Breathing-induced body surface displacement is on the order of millimeters. Taking 1 mm as an example, the phase change caused by it in a 120 GHz radar system is 1.6π rad, while it is 0.8π and 1.03π, respectively, in 60 GHz and 77 GHz systems. The high sensitivity due to the large phase angle variation is favorable for high accuracy measurement of TAA. Moreover, recent studies have suggested that high-frequency millimeter-wave radars are favored for better performance in high-precision measurements [29].

Finally, 120 GHz radar has a shallower penetration depth into the human body on the sub-millimeter scale compared to lower frequencies. Therefore, it mainly captures displacement information from the body surface, which facilitates the acquisition of a purer respiratory signal and reduces interference from other tissue movements inside the body. These characteristics make 120 GHz the preferred choice for chest and abdominal respiration monitoring.

#### 2.2.1. Narrow-Beam Antenna

A narrow-beam radar antenna is essentially a way to spatially avoid mutual interference between two radars. Narrow beam high-gain antennas also facilitate the accurate measurement of vital signs [30]. The mutual coupling of dual radars can be reduced by a narrow, half-power beamwidth (HPBW) with adequate radar spacing. Assuming that the HPBW is θ and the radar distance relative to the body is r, the diameter of the irradiated area formed by the radar on the body surface can be expressed as:

(3) $$d=2r*\tan\left(\frac{\theta }{2}\right).$$

The radar spacing should be greater than d, which can be expressed using the small-angle-approximation as:

(4) d≈rθ.

**Figure 3:**
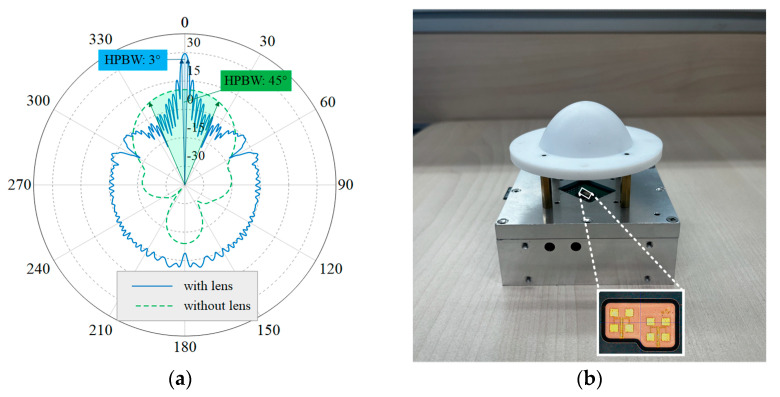
(**a**) The far-field radiation patterns of antennas with and without lenses. (**b**) A photograph of the lens antenna and the patch antenna feed. The inset shows the patch antenna within the radar chip package.

Silicon Radar’s TRX-120 was used as the 120 GHz radar front-end chip, employing antenna-in-package (AiP) technology to integrate a patch antenna within the chip package, thereby enhancing the directionality of the radar beam. Considering the patch antenna of the TRX-120 as a feed, a lens antenna was designed to further narrow the beam. A geometrical optics (GO) method was used for the lens design, followed by electromagnetic simulation using the FEKO 2020 software to optimize the profile. The lens is designed to be plano-convex and the feed is set at 50 mm from the apex of the convex surface of the lens to achieve a low profile. In this case, the feed is 22.8 mm from the lens plane and, as the HPBW of the patch antenna is about 45°, the radius of the lens needs to be larger than 9.4 mm to ensure that the lens receives the energy radiated by the feed effectively. The final design of the lens aperture is 50 mm, the material is low-loss polytetrafluoroethylene (PTFE), and its relative dielectric constant is 2.08 [31]. The far-field radiation pattern of the antenna is shown in Figure 3a. After increasing the lens antenna, the HPBW is changed from the original 45° to about 3°, which realizes the design goal of a narrow beam. The details of the patch antenna with the designed lens antenna are shown in Figure 3b. The chest and abdominal radars were placed at a distance of approximately 50 cm from the body. The spacing was 25 cm, which is much greater than d and meets the requirements for measurement.

#### 2.2.2. Radar Signal Design and Implementation

In addition to the use of narrow beams to achieve signal isolation in space, further design is required to avoid signals from one radar being picked up by another. Specifically, radar signals can be made more resistant to interference in both frequency and time dimensions. The FMCW radar front end continuously radiates linear Frequency Modulation (FM) signals, which are called chirps. The frequencies of the dual radars are completely separated with no overlap. However, this reduces the bandwidth of the radar to 1/2 of the original, thus losing half of the range resolution. Another approach is to segregate the radar signals in terms of time. As the radar’s chirp signal has a ramp time $T_{ramp}$ and an idle time $T_{idle}$ in a complete cycle, it is possible to control the dual radars so that one of them operates while the other one is idle. This approach results in a tiny, definite time delay between the dual radars that needs to be precisely controlled by hardware circuits. Despite the increased circuit complexity, the high range resolution facilitates better human target detection in complex environments. Therefore, the latter method is chosen in this study to design the radar signals.

The dual radar signals adopt the same configuration, and the ramp time should be less than the idle time to meet the condition of a completely independent radar working time. The parameters of the radar chirp signal, designed according to the respirometry application, are summarized in Table 1. The pulse repetition frequency (PRF) is the reciprocal of the chirp period, which is the sum of $T_{ramp}$ and $T_{idle}$. According to the Nyquist sampling theorem, the PRF of a radar should be greater than 1.2 Hz, which is twice the maximum respiratory rate of a normal person. Within the allowable range, increasing the PRF is beneficial for better estimation of respiration.

To realize the above dual radar configuration, an FMCW dual radar system based on a 120 GHz front-end MMIC was customized. Each of the dual radars has the same hardware, which consists of an RF front-end, an IF module, and a signal-processing module. The RF front-end adopts TRX-120 radar MMIC from Silicon radar GmbH whose internal voltage-controlled oscillator (VCO) is regulated by a phase-locked loop circuit (PLL) featuring of a frequency synthesizer (ADF4159C) and a microcontroller (STM32F303) on the IF board to enable the frequency sweep function. The MMIC includes phase shifters, mixers, and low-noise amplifiers (LNAs), and outputs I/Q quadrature IF signals. In addition to the PLL circuit, the IF module also implements the conditioning and sampling of IF signals. The LNAs are employed to reduce noise interference and amplify signals, and additional capacitors are added to filter out unwanted direct current (DC) bias components. The signal processing module adopts Xilinx’s ZYNQ-7020 system on chip (SOC), which integrates a dual core ARM (Advanced RISC Machine) Cortex-A9 processor and an FPGA (Field-Programmable Gate Array) xc7z020clg400-2 and has rich I/O interfaces. In order to achieve a precise dual radar delay function at low cost, we further utilized a signal-processing module with abundant hardware resources.

**Figure 4:**
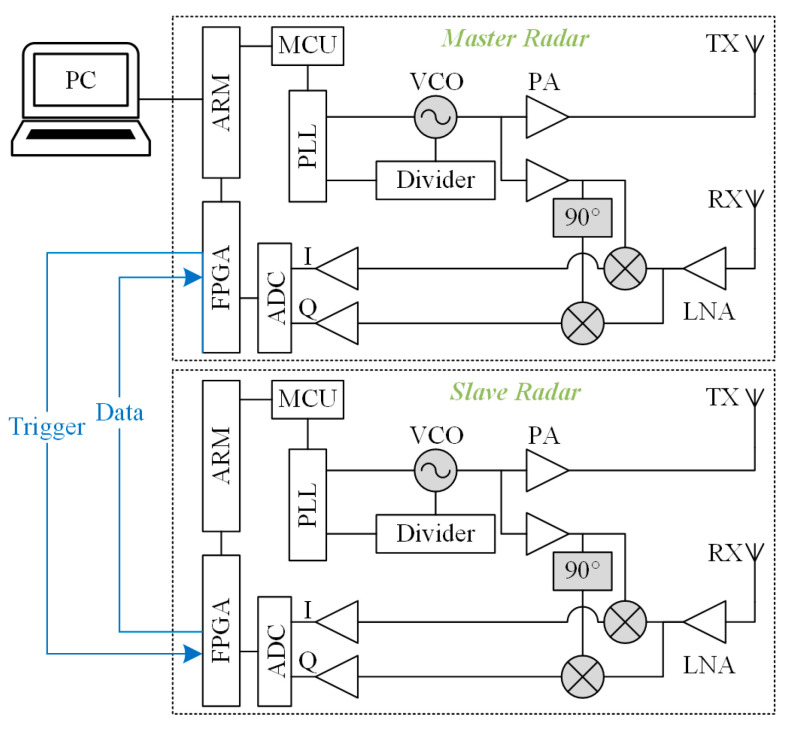
A circuit topology diagram for an interconnected dual radar system.

The topology structure of the dual radar interconnection is shown in Figure 4. One of the dual radars was set as the master, and the other was set as the slave. Both radars are controlled by a high-level trigger signal to start sweeping. A set of FPGA pins is reserved for communication for each of the two radars and is connected by wires. Since the spacing between the chest radar and the abdominal radar does not exceed the length of the human torso—i.e., about 40 cm—the transmission delay of the signals on the wires is negligible. The host pulls up its own trigger signal level according to the programmed period and pulls up the slave’s trigger signal level after $T_{delay}$. The slave detects the high trigger level, and controls the sweep and analog-to-digital converter (ADC) sampling, and sends the data to the host. The host summarizes its own data and the slave’s data and uploads them to the PC.

The current BOM cost of the customized dual radar system is approximately USD 700: USD 100 for the pair of 120 GHz MMIC; USD 400 for the Zynq-7020 SoC development board; USD 140 for the PTFE lenses; and ~USD 60 for remaining components, PCB fabrication, and housing. Once the SOC is migrated from the dev kit to a purpose-designed board and produced in volume, the total cost is projected to drop by 40–50% (to approximately USD 300–450).

In summary, the dual radar is a complete system and is only used by the host radar and the upper computer for communication. An accurate and reliable delay function is implemented through hardware circuits, providing support for high-precision and multi-dimensional breathing pattern measurement. The cost of the system is economically feasible for home and clinical penetration.

### 2.3. Breathing Pattern Estimation Algorithms

The respiratory waveform contains rich information that can provide a basis for disease diagnosis. Merely the calculating respiratory rate for respiratory distress diagnosis is far from sufficient. Therefore, the focus of this article is on how to use a dual radar to extract more information about breathing pattern from chest and abdominal movements. In order to accurately estimate the respiratory pattern, a series of signal processing algorithms are proposed to reconstruct the respiratory waveform, suppress motion interference and calculate the thoracic–abdominal phase angle.

#### 2.3.1. Respiratory Waveform Reconstruction

Reconstruction of the respiratory waveform from the radar signal begins with phase demodulation. To achieve simple and efficient phase demodulation, an arctangent method with phase unwrapping [32] is used in this study. Specifically, after FFT of the I/Q complex signals, the spectral peak index is selected as the range bin where the human target is located, and the phase is calculated using the arctangent method from its real and imaginary parts. Finally, the phase sequence extracted from multiple consecutive chirp signals is subjected to unwrapping processing. For two adjacent phase values, φ(n) and φ(n+1), if $\varphi \left(n+1\right)-\varphi \left(n\right)>\pi$, $\varphi \left(n+1\right)=\varphi \left(n+1\right)-2\pi$; if $\varphi \left(n+1\right)-\varphi \left(n\right)<-\pi$, $\varphi \left(n+1\right)=\varphi \left(n+1\right)+2\pi$. The phase sequence is then multiplied by the dimensional conversion factor $\frac{\lambda }{4\pi }$ to obtain the displacement signal.

Another key aspect of waveform reconstruction is filtering out heartbeat clutter and other noises while retaining important features, such as peaks, without phase delay, thus providing a reliable basis for the subsequent extraction of respiratory parameters and the calculation of the chest–abdominal phase difference.

**Figure 5:**
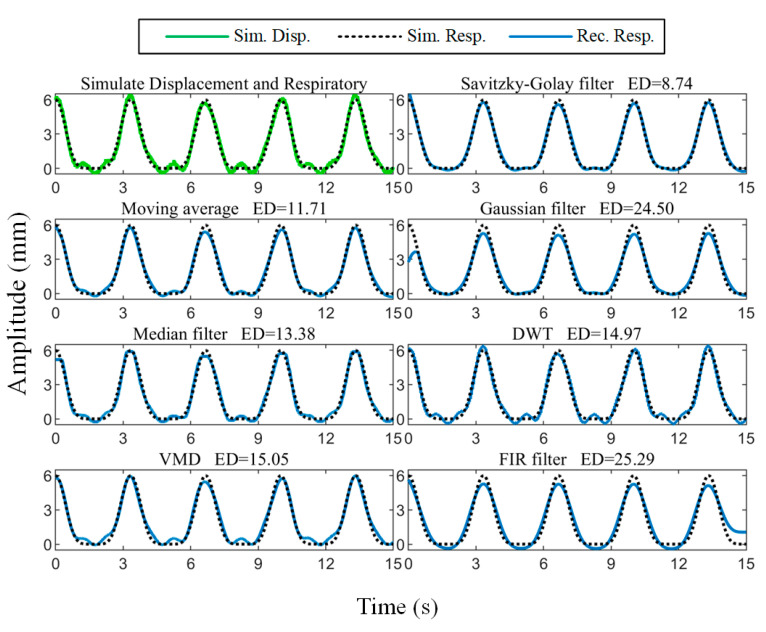
A comparison of respiratory waveforms reconstructed using Savitzky–Golay filtering, moving average, Gaussian filtering, median filtering, wavelet decomposition, VMD, and FIR filtering algorithms.

Simulation experiments were performed to compare the performance of common filtering methods without phase distortion. The respiration displacement is modeled according to [33] and expressed as

(5) $$x_{r}\left(t\right)={A_{r}cos}^{2n}\pi f_{r}t,\;\;\;\;n\in Z^{+}$$

where $A_{r}$ is the respiratory amplitude, $f_{r}$ is the respiratory frequency, and n is the total number of respiratory fundamental and harmonic frequencies. Correspondingly, the heartbeat displacement is modeled according to [34] as

(6) $$x_{h}\left(t\right)=A_{h}sin(2\pi f_{h}t)$$

where $A_{h}$ is the heartbeat amplitude, and $f_{h}$ is the heartbeat frequency. The body surface displacement signal was simulated according to (2) and Gaussian noise with an SNR of 30 dB was added, as shown in Figure 5. The respiratory amplitude was set to 6 mm with a frequency of 0.3 Hz, and the heartbeat amplitude was 0.4 mm with a frequency of 1 Hz.

The comparison of the reconstructed respiratory waveform results obtained using the moving average [35], median filtering [36], Gaussian filtering [37], zero-phase low-pass FIR filtering (LPF) with a cutoff frequency of 0.6 Hz, VMD [38], discrete wavelet transform (DWT) [39], and the Savitzky–Golay filtering algorithm is given in Figure 5. To quantitatively characterize the similarity between the reconstructed results and the setup ground truth, we calculated the Euclidean distance between the two:

(7) $$d\left(\hat{y},y\right)={\left\|\hat{y}-y\right\|}_{2}$$

where $\hat{y}$ and y denote the reconstructed respiratory waveform and the simulated respiratory signal, respectively, and ${\left\|\cdot \right\|}_{2}$ denotes the $L_{2}$ norm. It can be seen that the Savitzky–Golay filtering result has the smallest Euclidean distance from the simulated signal.

In addition, the Savitzky–Golay filtering algorithm has two hyperparameters: polynomial order k and window length l. The third-order polynomial and the window length of $1.4*f_{s}$ ($f_{s}$ is the signal sampling rate) are appropriate, as determined through simulation experiments with different parameters.

#### 2.3.2. Motion Interference Suppression

It is difficult to avoid motion artifacts from unconscious physical activity during prolonged respiratory monitoring. Displacements generated by random body movements can severely interfere with the respiratory signal of interest and significantly reduce the accuracy of detection. In order to obtain highly accurate respiratory parameters, motion interference must be eliminated.

Interferences caused by body movements can be categorized into two types: baseline shifts caused by slow movements and large, abrupt changes caused by sudden body movements. For the former, this work uses VMD [40] to decompose the signal into several intrinsic modal functions (IMFs), and the IMF reflecting the trend is subtracted from the original signal to suppress baseline drift. The VMD algorithm extracts characteristics from the signal itself and is well-suited for dealing with nonlinear and non-stationary signals.

Sudden body movements such as coughing and sneezing are more random in nature. They have a greater impact on respiratory parameters and are therefore the focus of this study.

1. DTW Similarity Estimation Algorithm

The DTW algorithm [41] is a method that can compute the similarity of time series of different lengths, and our identification of interfered data segments is based on the realization that it is less similar to normal breathing. The DTW algorithm improves upon the Euclidean distance that calculates the similarity of time series of different lengths. The core idea of the algorithm is to find the optimal matching of two sequences to make them nearest based on dynamic programming. For two respiratory cycle data segments X and Y with lengths m and n, respectively, the Manhattan distance is used to define the distance between points in the two sequences:

(8) $$d\left(x_{i},y_{j}\right)=\left|x_{i}-y_{j}\right|$$

where $x_{i}$ and $y_{j}$ are the i-th and j-th data points in X and Y, respectively. Then, an m×n cost matrix C is constructed, and each element C(i,j) in the matrix represents the cumulative distance between the first i points of X and the first j points of Y. The recurrence formula for the cost matrix C is

(9) $$C\left(i,j\right)=d\left(i,j\right)+min\left[C\left(i-1,j\right),C\left(i,j-1\right),C\left(i-1,j-1\right)\right]$$

where i>1,j>1. Path backtracking from the lower right corner of the cost matrix C(m,n) is performed to find the path with the minimum cumulative distance and finally, the DTW distance is obtained:

(10) $$DTW\left(X,Y\right)=C(m,n)$$

**Figure 6:**
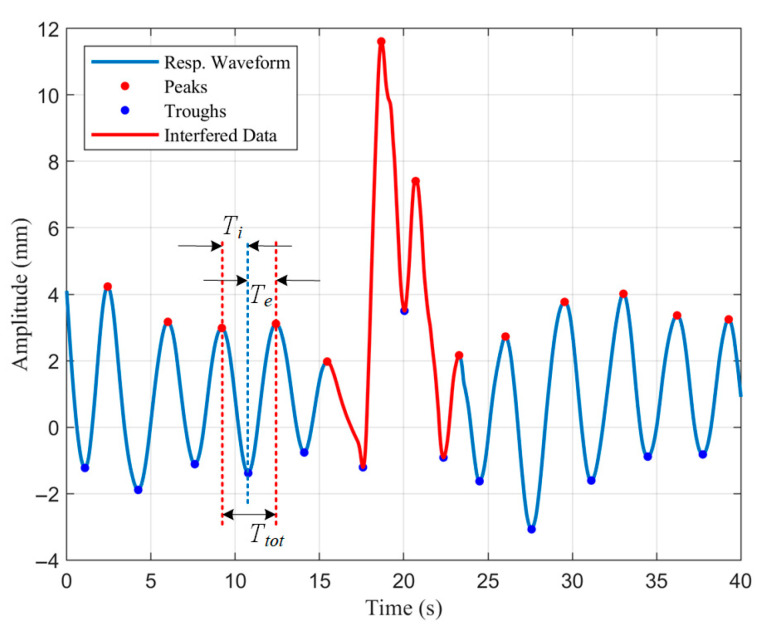
The identification and removal of interfered data segments.

The Savitzky–Golay filtered respiratory waveform is smooth enough to easily achieve peak detection by finding local maxima. To avoid false detection, we set the minimum inter-peak distance to 1.5 s, corresponding to a respiratory frequency of 40 bpm. The sign of the waveform data was flipped and peak detection was performed again to determine the location of the troughs. Numerous respiratory parameters can be determined using the peaks and troughs as characterization points. The inspiratory time $T_{i}$, expiratory time $T_{e}$, total breath cycle time $T_{tot}$, and inspiratory time fraction $T_{i}/T_{tot}$ were derived from the time intervals between consecutive peaks and troughs in the respiratory waveform, as shown in Figure 6. The respiratory rate is the reciprocal of the respiratory cycle and usually needs to be multiplied by 60 to change the units from Hz to bpm. The respiratory amplitude is the distance between peaks and troughs.

At the beginning of the test, subjects were asked to hold a relaxed, steady breath for a few seconds, allowing a breathing cycle to be selected as a reference signal $X_{ref}$. The DTW algorithm was utilized to calculate the similarity of all respiratory cycle data segments within the time window to the reference signal to obtain a similarity vector S. The element $s_{i}$ in vector S represents the distance between the ith respiratory cycle and the subject’s normal respiration. The larger its value, the greater the likelihood that the data segment is interfered with, and vice versa.

2. Adaptive Classification Method for DTW-Derived Similarity

To solve the problem wherein it is difficult to setup a differentiation criterion for the elements in the similarity vector S obtained by DTW computation, this study proposes the use of the K-means clustering algorithm [42] to perform adaptive binary classification for the elements in S. The K-means is a method used to determine the allocation of clusters by minimizing the sum of the squares of the distances of the data points within each cluster to the mean of that cluster, and its objective function can be expressed as

(11) $$SSE=\sum_{j=1}^{k}\sum_{x\in S_{j}}{\left\|x-c_{j}\right\|}^{2}$$

where SSE is the sum of squared errors; k is the number of clusters which is set to 2; $S_{j}$ is the set of all data points in the j-th cluster; and $c_{j}$ is the cluster center of the j-th cluster.

The initial clustering centers chosen randomly by the K-means algorithm may cause the algorithm to converge to a local optimum solution, affecting the accuracy of the results. Leveraging the prior knowledge that the DTW distances of breath cycles with motion artifacts are significantly larger than those of normal breath cycles, we selected the DTW distance of a reference breath cycle and the maximum value in the similarity vector S as the initial cluster centers for the two clusters:

(12) $$C=\left\{C_{1}{,\;C}_{2}\right\}=\left\{S_{ref},max(S)\right\}$$

Subsequently, data point assignment and cluster center update were performed:

(13) labelsi=arg mincj∈Csi−cj

(14) $$c_{j}=\frac{1}{\left|S_{j}\right|}\sum_{s_{i}\in S_{j}}s_{i}$$

where $S_{j}$ is the set of all data points belonging to Cluster *j* and $\left|S_{j}\right|$ is the size of the set. We then iteratively optimized:

(15) $$if\left\|c_{j}^{t+1}-c_{j}^{t}\right\|<\epsilon \;or\;t=T,stop$$

Sudden movement interference can be described as a low-probability, short-lived stochastic event, consequently leading to affected data segments that are significantly shorter in duration than typical data. Therefore, the cluster with smaller amounts of data are considered interfered with, and the respiration cycle data corresponding to the elements in it are set to zero. Moreover, when there is no interference within the processing window, S is still categorized into two clusters. Therefore, we additionally add special case handling to enhance robustness. If the number of elements in the interfered cluster obtained by K-means is more than one-third of all elements, all respiratory cycles within the window are considered to be in the normal case:

(16) $$if\;\left|S_{j}\right|>\frac{n}{3},\;then\;label\;all\;s_{i}\in S_{j}\;as\;normal.$$

To further improve robustness and avoid the missed detection of abnormal breathing patterns, additional processing is added after the above steps. As the recognition of motion interference is based on similarity comparisons, apneas may be misclassified as motion artifacts. Fortunately, apnea has another characteristic, which is a significant reduction in the amplitude of the respiratory waveform over a period of time. Labeling of apnea events is performed by calculating whether the decrease in amplitude exceeds 30% and lasts longer than 10 s [43].

#### 2.3.3. Pathological Breathing Pattern Recognition

**Figure 7:**
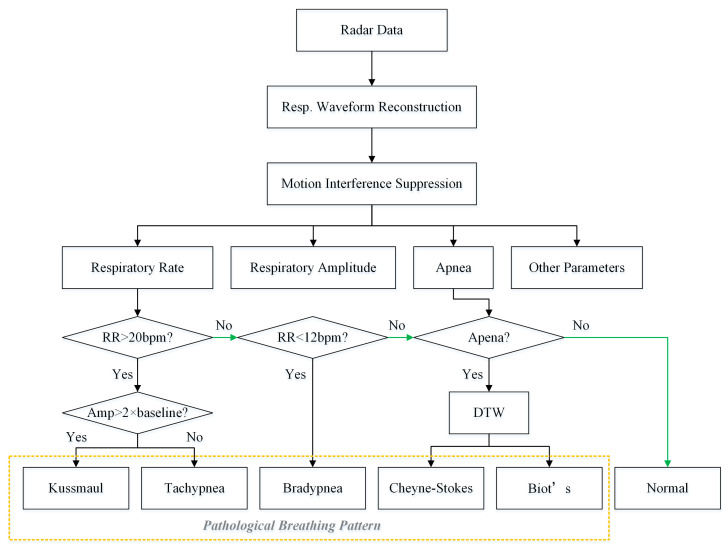
The pathological breathing pattern recognition flowchart.

Based on the detected respiratory rate, respiratory amplitude and apnea, five pathological breathing patterns were investigated in this paper, including tachypnea, bradypnea, Kussmaul, Cheyne–Stokes, and Biot’s respiration. The flowchart used to detect abnormal breathing patterns is shown in Figure 7. Among them, tachypnea, bradypnea, and Kussmaul breathing do not exhibitapnea and can be discriminated using the respiratory rate and respiratory amplitude. Cheyne–Stokes and Biot’s breathing are both accompanied by apnea and are further recognized using the adaptive DTW algorithm to compute the similarity with typical templates.

During the motion interference suppression phase, the similarity between respiratory cycles was calculated using adaptive DTW to identify interfered data segments. Moreover, the algorithm can also recognize pathological breathing patterns. Specifically, a template library of typical Cheyne–Stokes and Biot’s respirations was first established. Then, the radar data were appropriately segmented, the DTW distances of the two templates were computed separately, and pathological respiratory patterns were recognized by comparing them with a preset threshold.

**Figure 8:**
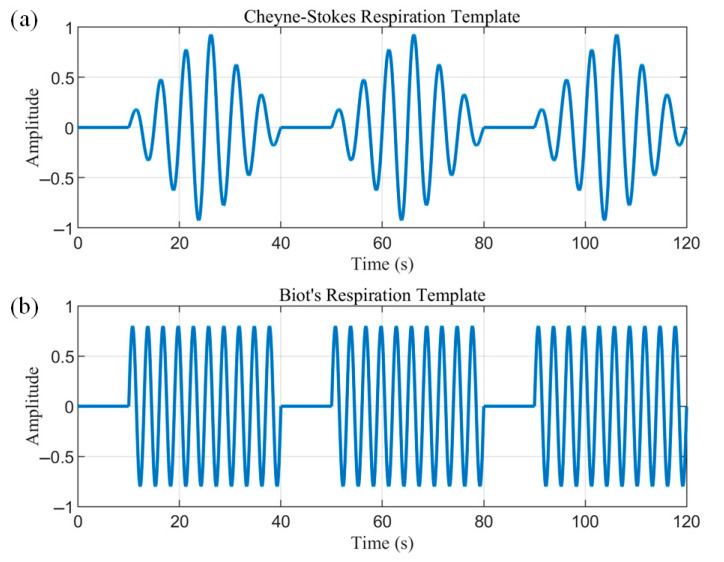
(**a**) The Cheyne–Stokes respiration template. (**b**) Biot’s respiration template.

Cheyne–Stokes respiration (CSR) is a respiratory pattern in which the respiratory amplitude periodically crescendos and decrescendos with regular apneas at the end of each cycle. Biot’s respiration is characterized by regular breathing with sudden apneas. Based on the characteristics, two templates were created, as shown in Figure 8. The templates contain three cycles each, covering the complete pattern of abnormal respiration and avoiding the randomness of a single cycle.

The interception of the radar data also began with an apnea and ended at the moment of the fourth apnea so that the data segment matched the template. The data segments were then normalized for magnitude, and their normalized DTW distance from the two templates was calculated. The smaller of the two distances was compared to the corresponding threshold value T. If D was less than T, it was recognized as the corresponding pathological breathing pattern, where the threshold T was empirically set to 0.3.

#### 2.3.4. Thoracoabdominal Phase Angle Measurement

Thoracoabdominal asynchrony (TAA) is an important indicator of the respiratory status of the subject, indicating the presence of respiratory impairment and respiratory distress. It provides a method for clinical measurement of respiratory distress in patients with COPD, as well as infants and children. Due to the possible non-sinusoidal nature of the respiratory waveform, the cross-correlation technique was chosen to measure TAA in this study

(17) $$Xcor\left(\tau \right)=\int_{-\infty }^{\infty }THX(t)\cdot ABD(t+\tau )dt$$

where THX denotes the thoracic respiratory waveform, ABD denotes the abdominal respiratory waveform and τ denotes time offset. The maximum value of the result of the cross-correlation function indicates the moment when the two signals are most similar, and by denoting the time offset at this point as $\tau_{max}$, the phase angle of the thoracic and abdominal motions can be calculated as follows:

(18) $$\varphi =2\pi \frac{\tau_{max}}{T_{cycle}}$$

**Figure 9:**
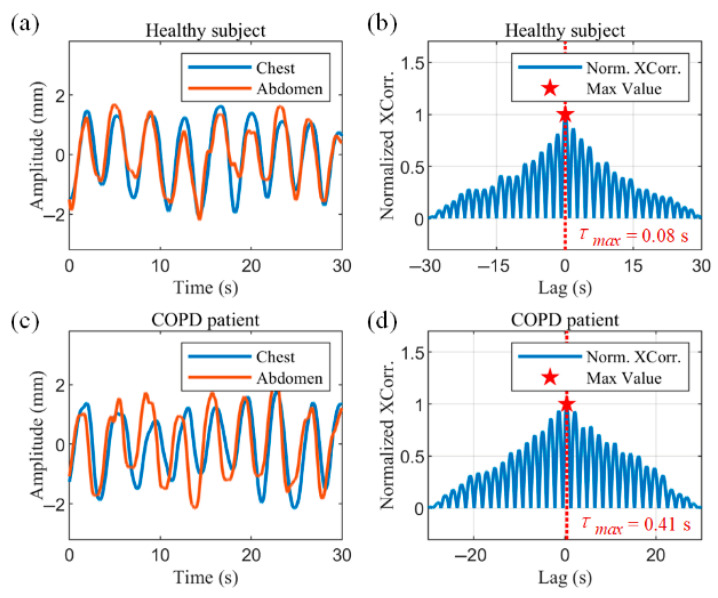
Measurements of thoracoabdominal asynchrony in a healthy subject and a COPD patient. (**a**) Respiratory waveforms from the chest and abdominal radars of the healthy subject. (**b**) Normalized cross-correlation results of chest and abdominal movements in the healthy subject. (**c**) Chest and abdominal respiratory waveforms of the COPD patient. (**d**) Normalized cross-correlation results of chest and abdominal motion in the COPD patient.

Figure 9a,b show the radar-captured chest–abdominal respiratory waveforms, and the normalized chest–abdominal cross-correlation results for a healthy subject, respectively. The chest–abdominal waveforms show good synchronization. The maximum normalized cross-correlation result is marked with a pentagram in Figure 9b. The horizontal coordinate of the point is 0.08 s, corresponding to a phase angle of 8.11°, indicating a minimal time delay between thoracic and abdominal movements. Figure 9c,d show the measurements in COPD patients. Asynchrony of motion can be observed in the waveforms in Figure 9c. The abdominal motion is earlier than the chest motion. The horizontal coordinate of the maximum of the normalized cross-correlation results in Figure 9d represent a time delay of 0.41 s. According to (18), the thoracoabdominal phase angle is 41.58°, where $T_{cycle}$ is the mean value of the respiratory cycle within the data window.

## 3. Experimental Setup

The experiments used a customized 120 GHz FMCW dual radar system as described previously. The system performed the transmitting, receiving and mixing of RF signals, as well as the conditioning and sampling of IF signals. It also performed the signal processing steps of human target localization, phase extraction and unwrapping. The measured chest and abdomen displacement data were then sent to a PC for further processing.

**Figure 10:**
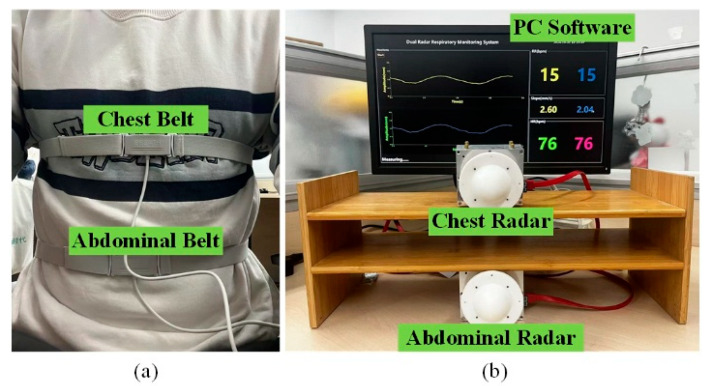
(**a**) The chest and abdominal respiratory belts used as a reference. (**b**) The dual radar system with radar hardware and PC software. The software was used for radar control and to display real-time respiratory waveforms and parameters.

The position of the dual radar system directly affects the measurement results, so the distance and angle of the radar relative to the human body need to be considered. According to (4), the distance between the radar and the human body affects the area of the beam irradiation region. The distance should be shortened to better distinguish the chest and abdominal movements. As the radar sensor has a narrow antenna beam, the angle between the radar and the human body should be adjusted such that the angle of incidence is close to zero degrees to ensure that the echo can be picked up by the receiver. The dual radars were placed directly facing the chest and abdomen, spaced 25 cm apart and both 50 cm from the body. The experimental setup is shown in Figure 10. The chest and abdominal respiratory bands used as reference are shown in Figure 10a. Figure 10b shows the dual radar system with the custom-developed PC software used to control and display real-time waveforms with respiratory parameters.

We recruited 35 healthy subjects (21 males and 14 females, aged between 21 and 58 years and weighing between 46 and 90 kg), 2 patients with chronic obstructive pulmonary disease (COPD) and 1 patient with heart failure. The first 33 healthy individuals and the COPD patient each underwent a test of about 15 min, and the last 2 healthy individuals and the heart failure patient underwent a sleep experiment of about 8–12 h. We numbered the healthy subjects as 1–35, the COPD patient as 36–37, and the heart failure patient as 38. Written informed consent was obtained from all subjects. Subjects were dressed in normal attire, and all experiments were performed in a seated position, with their backs straight against a chair to minimize body movement. The performance of the dual radar system was evaluated using two respiratory monitoring belts strapped to the chest and abdomen, respectively, as reference signals.

## 4. Experimental Results

### 4.1. Chest Breathing and Abdominal Breathing

None of the first 35 subjects suffered from respiratory disease, and each subject sat still for the test. Due to differences in breathing habits, the data from these subjects showed both thoracic and abdominal breathing patterns.

**Figure 11:**
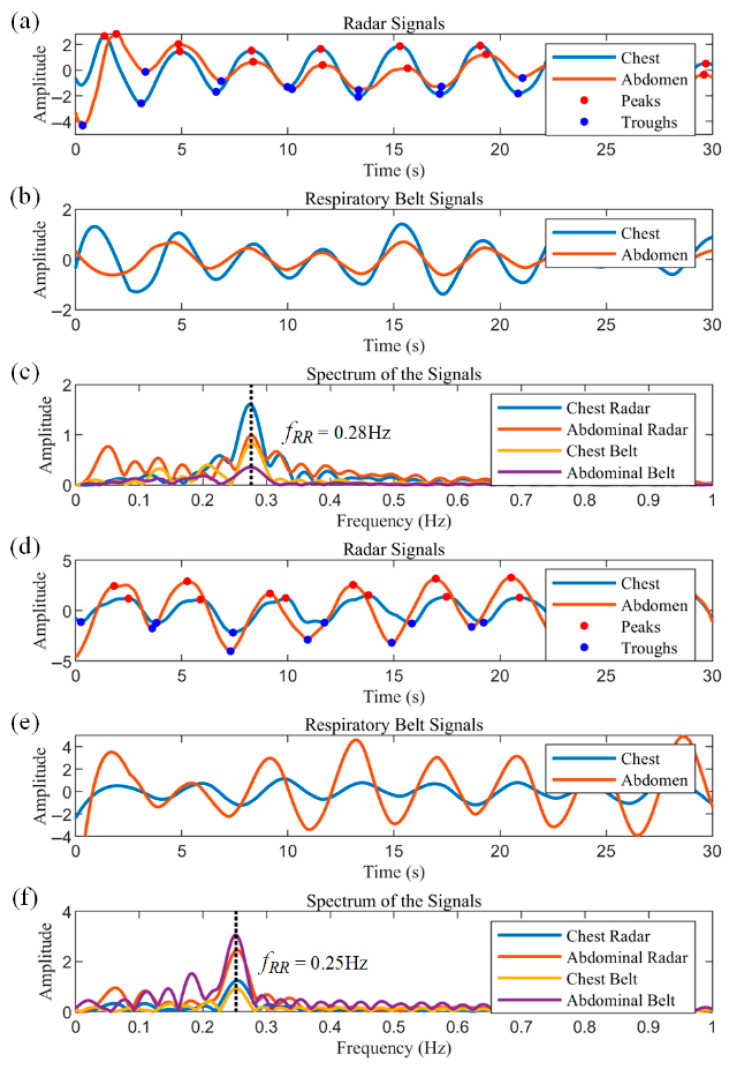
Radar and respiratory belt measurements in healthy subjects with chest as well as abdominal breathing. (**a**–**c**) Radar signals, respiratory belt signals and signal spectra for a chest breathing subject, respectively. (**d**–**f**) are the measurements for an abdominal breathing subject. The dashed lines in (**c**,**f**) indicate the peaks corresponding to the respiratory frequency.

Figure 11a–c illustrate 30 s segments extracted from the data of Subject 1. Figure 11a shows the respiratory waveforms of the chest radar and the abdominal radar, which show that the amplitude of the chest radar is larger than that of the abdominal radar. The peaks and troughs of the respiratory waveforms are labeled as red and blue dots, respectively. Based on these characteristic points, the inspiratory and expiratory phases can be easily classified, and in turn, the inspiratory time, expiratory time and respiratory period of each cycle can be obtained directly. The data from the chest and abdominal respiratory belts are displayed in Figure 11b, where the signal amplitude of the chest respiratory belt is similarly larger than that of the abdominal respiratory belt, and the data from the two sensors show good agreement. Figure 11c shows the frequency spectra of the radar and respiratory belt data, with peaks at approximately 0.28 Hz for all sensors, corresponding to 16.8 breaths per minute.

Subject 2 has a fitness habit of using abdominal breathing to stabilize the core during strength training, so his test data show a distinct abdominal breathing signature. Figure 11d–f show the test data of Subject 2, all of which are identical to Figure 11a–c. As can be seen in Figure 11d,e, the amplitude of the abdominal respiratory waveform is much larger than that of the chest, as evidenced by the peaks representing the respiratory components in the spectrum of Figure 11f.

### 4.2. Motion Interference Suppression Verification

**Figure 12:**
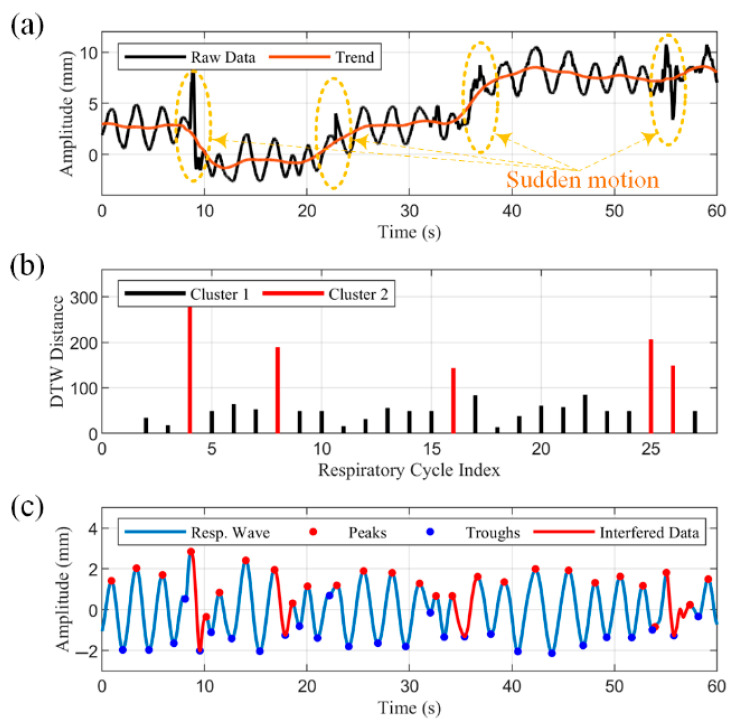
(**a**) Interfered raw data, including slow body movements and four bursts of movements. (**b**) DTW distances for each respiratory cycle from the first respiratory cycle, and the results after clustering the DTW distances. (**c**) Reconstructed respiratory waveforms with interfered data segments identified by the proposed method.

To verify the reliability of the proposed motion interference suppression algorithm, we show a 60 s segment of data in Figure 12. The solid black line in Figure 12a indicates the raw radar data in which low-frequency trends, periodic breathing signals, and high-frequency noise can be observed. The data were decomposed into three IMFs using the VMD, and the IMF with the lowest frequency represents the motion trend term—as shown by the solid red line—which reflects the slow, unconscious motion of the subject’s body during the test period, resulting in a baseline drift of the data. The subject’s body approached the radar around the 10th second and gradually moved away thereafter. The dashed yellow box indicates the interference caused by sudden body movements, where subjects showed sudden movements, such as coughing or sneezing, around the 9th, 17th, 34th, and 55th seconds, respectively. The vertical line in Figure 12b shows the DTW distance of the first respiratory cycle from all respiratory cycles after smoothing the data and decomposing them into independent respiratory cycles via feature points. There are some cycles for which the results are significantly larger than others, which means that there is a large difference between these cycles and the first cycle. After binary classification of DTW distances, the results are distinguished in black and red. Figure 12c shows the reconstructed respiratory waveform with interfered data segments, indicated by solid blue and red lines, respectively. These disturbed periods were identified according to the index of Cluster 2 in Figure 12b and correspond well to the moments in Figure 12a, where sudden movements occur. The reconstructed respiratory waveforms have clear feature points of peaks and troughs, and the mean value shifts to near zero after subtracting the motion trend.

To quantify the effect of motion interference suppression, the respiratory SNR was calculated by integrating the power spectral density (PSD) before and after applying the proposed suppression method. The mean ± SD SNR improved from −7.34 ± 1.21 dB to −2.98 ± 0.87 dB, corresponding to an enhancement of 4.36 dB. Under identical interference, EMD and wavelet soft-thresholding (sym8, level 5, the universal threshold) achieved 1.97 ± 0.42 dB and 2.34 ± 0.55 dB SNR enhancements, respectively. Regarding the respiration rate RMSE, the error reduction rate reached 31.4%, 37.8%, and 58.1% for EMD, wavelet, and our proposed method, respectively. The proposed algorithm detects and recognizes interfered data well, demonstrating the ability of the system to suppress interference effects.

### 4.3. Pathological Breathing Pattern Detection

The recognition of pathological respiratory patterns relied on the detection of apnea, so apnea experiments were performed first. The experiment recorded data from a subject who held his breath to simulate apnea, an abnormal breathing pattern, and who coughed during the period to see if the proposed algorithm could differentiate between the two scenarios.

**Figure 13:**
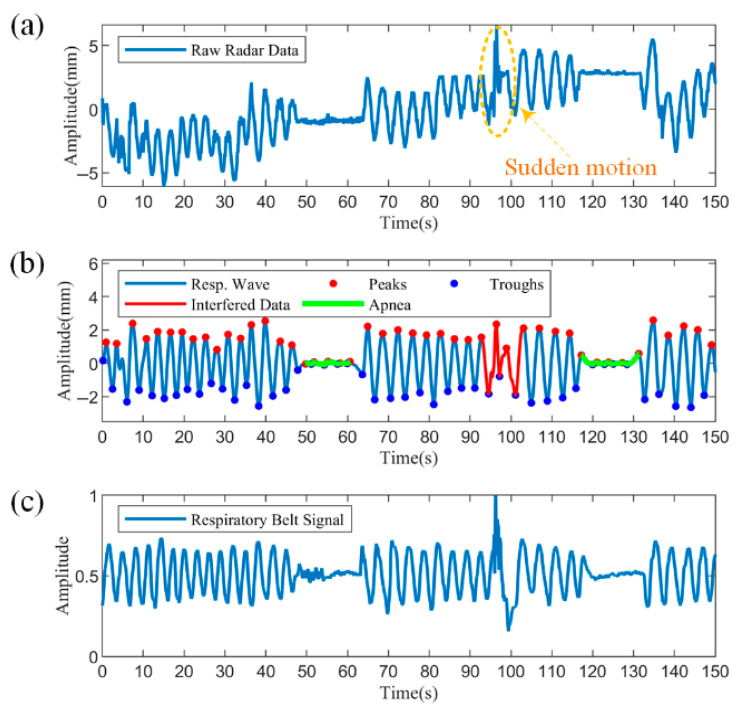
Measurements of abnormal breathing patterns. (**a**) Raw radar data accompanied by one sudden body motion and two apneas. (**b**) Processed respiratory waveforms with the detection of motion interference and apneas. (**c**) Simultaneously measured respiratory belt signal.

Figure 13 shows the segment containing apnea with motion disturbances, with a data length of 150 s. Figure 13a shows the raw radar data, from which the body movement trend can be observed. From approximately 48 to 63 s and 117 to 131 s, there is a significant decrease in amplitude due to apnea. At approximately 94 s, a sudden coughing movement resulted in an irregular change in the data waveform, marked by the dashed yellow box. Figure 13b demonstrates the results processed by the proposed algorithm. The reconstructed respiratory waveforms are uniformly distributed around the zero value after filtering and detrending. The disturbed data can be easily detected after utilizing the similarity to the respiratory cycle, as shown by the solid red line. Further, two apneas were accurately identified based on the decision rule of a 90% reduction in amplitude versus duration over ten seconds, as shown by the solid green line. Figure 13c illustrates the synchronized measured respiratory band data. From the respiratory band data, both the sudden body movement and the two apneas are clearly observed and have largely overlapping coverage times with the radar data. Compared to the raw radar data in Figure 13a, the respiratory band data do not tend to slow body movements, because the respiratory bands are strapped to the body, maintaining a slow movement in synchronization with the body. Overall, the processed respiratory waveforms are more similar to the breathing band reference signal and can detect and differentiate abnormal respiratory patterns from motion disturbances, proving the effectiveness of the proposed method.

**Figure 14:**
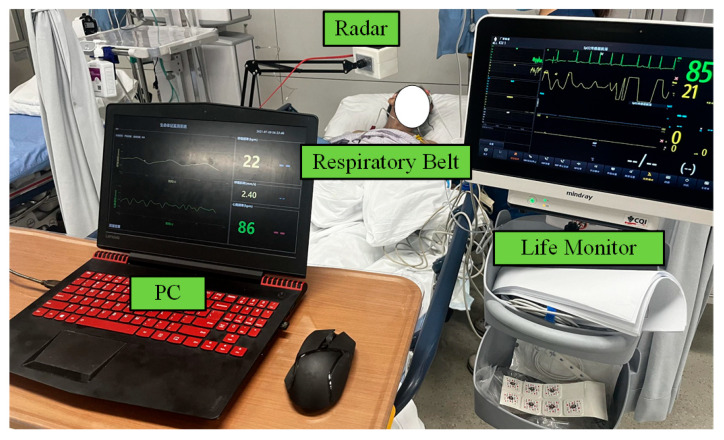
The experimental setting for a patient with heart failure.

To further validate the feasibility of the proposed method for recognizing pathological respiratory patterns, an experiment was conducted on a heart failure patient in a clinical setting, as shown in Figure 14. The experiment was conducted in a hospital room with the radar fixed using a stand at a distance of about 50 cm from the body. The heart failure patient was monitored with an abdominal respiratory belt as he relied more on diaphragmatic breathing due to impaired lung function and could not tolerate the chest belt. Twelve hours of continuous recording was performed on this patient to monitor sleep.

**Figure 15:**
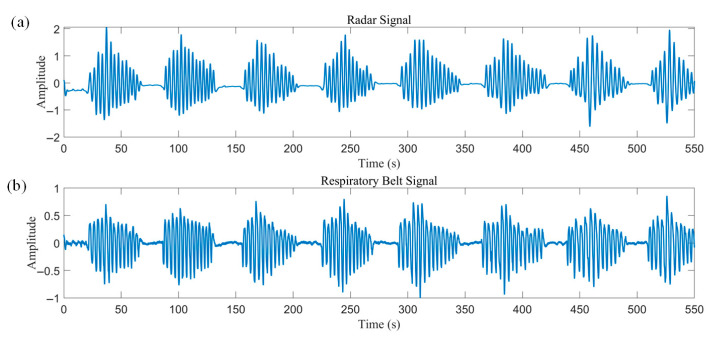
CSR during sleep—550 s of radar with respiratory belt data. (**a**) Radar data. (**b**) Simultaneously measured respiratory band data.

The patient experienced multiple CSRs during sleep, ranging from ten minutes to nearly an hour each. Figure 15 illustrates a set of 550 consecutive seconds of radar and respiratory belt data, which showed good agreement with a correlation coefficient of 0.91.

**Figure 16:**
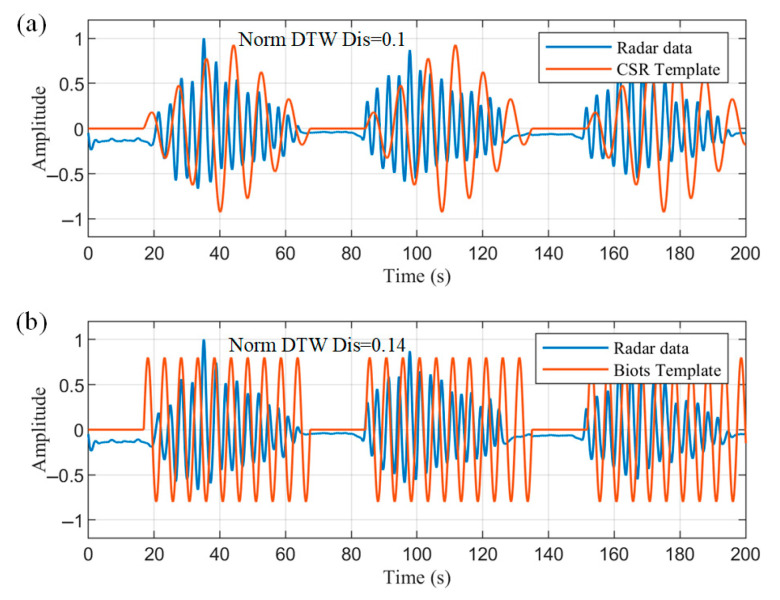
The normalized DTW distances of intercepted radar data with (**a**) CSR template (**b**) Biot’s template, respectively.

The detected apnea onset location was used as a feature, and three consecutive crescendo–decrescendo cycles were intercepted from the radar data and normalized, as shown in Figure 16. The normalized DTW distances between the data and the two templates were then solved for separately. DTW does not require equal-length sequences, and distances can be calculated even between 200 s radar data and 120 s templates. This process dynamically warps the templates to align with the radar data, as approximated by the orange curves in Figure 16. The calculated averaged normalized DTW distances of the radar data from the CSR template and Biot’s template are 0.1 and 0.14, respectively. Thus, the data are more similar to the CSR template and meet the less-than-threshold condition to be correctly identified as a CSR pathological breathing pattern.

### 4.4. Paradoxical Respiration Detection

For this experiment, subject 36 and 37 were asked to adopt the same posture as the first 35 subjects. The subjects had severe COPD and suffered from respiratory muscle fatigue due to chronic overuse of the diaphragm. This affected the coordination of thoracic and abdominal movements, leading to paradoxical breathing.

**Figure 17:**
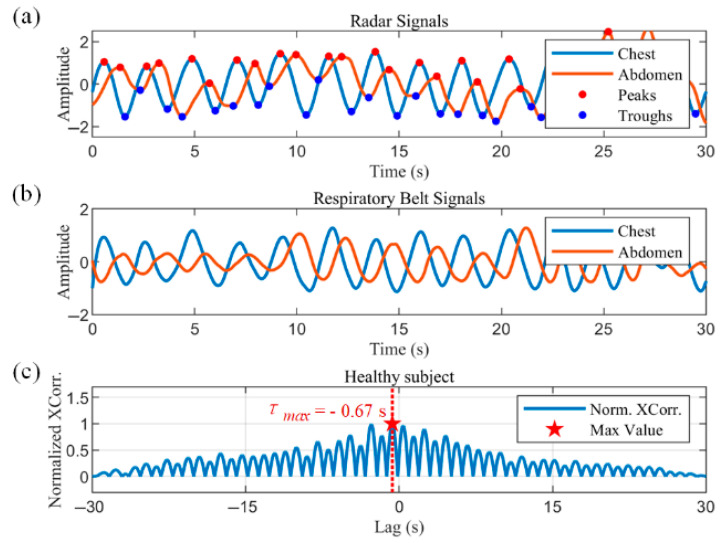
Measurements in patients with COPD. (**a**) Radar signal in the chest and abdomen. (**b**) Respiratory belt signal. (**c**) The normalized cross-correlation results of radar signals.

Asynchrony of the thoracic and abdominal movements can be clearly seen in Figure 17. Figure 17a illustrates the respiratory waveforms measured by the thoracic and abdominal radars, which differ in amplitude and shape, representing differences in the manner of movement at the two locations. The data have approximately the same frequency and roughly opposite phases, representing that the subject had respiratory effort in both the thorax and the abdomen during each respiratory cycle, but the movement was almost the opposite direction. The results of respiratory belt measurements in Figure 17b also reflect such a trend. The chest–abdominal phase time difference calculated using the proposed method is shown in Figure 17c, which corresponds to a chest–abdominal phase angle of approximately 120.6°. Comparison with the breathing patterns of healthy subjects in Figure 11a,b shows that COPD patients breathe more shallowly and quickly, with poorly coordinated thoracic and abdominal movements, which may be due to airflow limitation resulting from pathophysiologic changes in the patients. Among the two COPD patients enrolled, $T_{i}/T_{tot}$ was significantly lower (0.29 ± 0.02) than that of the 35 healthy subjects (0.41 ± 0.03), consistent with the airflow limitations characteristic of obstructive disease.

It was demonstrated that the cross-correlation method can measure the thoracic–abdominal phase angle to characterize paradoxical respiration, even though the amplitude and shape of the respiratory waveforms of the thoracic and abdominal radars differ in the case of non-sinusoidal respiratory waveforms.

## 5. Discussion

### 5.1. System Performance Evaluation

To illustrate the performance of the proposed system in estimating breathing patterns, data from all subjects are quantitatively analyzed in this section. The basic information and health status of all subjects are summarized in Table 2.

Table 3 summarizes the statistical performance evaluation of the radar signals and the reference breathing belt signals, where all data were excluded from the abnormal segments. The results showed a high correlation between radar and respiratory bands in both the chest and abdomen. The respective average correlation coefficients for the chest and abdomen were 0.92 across all subjects. The accuracy of the respiratory rate was measured using the root mean square error (RMSE):

(19) $$RMSE=\sqrt{\frac{1}{n}\sum_{i=1}^{n}{\left(y_{i}-\hat{y_{i}}\right)}^{2}}$$

where n is the number of observations, $y_{i}$ is the i-th true value, and $\hat{y_{i}}$ is the i-th measured value. The RMSE of the respiratory rate was about 0.8 for both the chest and the abdomen. As for the TAA measurements, the mean standard deviation (SD) is 4.3° and the mean absolute error (MAE) amounted to 3.4°. The MAE of 5.8° for subject 4 is attributed to a slight chair rotation during the experiment, which shifted the radar footprint away from the optimal position. An inspection revealed a 30% lower raw displacement amplitude and, consequently, a lower SNR, increasing phase estimation uncertainty. In a follow-up measurement with the antennas realigned to 0° incidence, the TAA error was reduced to 3.1°. In the test with paradoxical breathing, the proposed system still showed superior performance, demonstrating that the dual radars work without mutual interference and that the system can attain noncontact accurate measurements of thoracic and abdominal movements.

**Figure 18:**
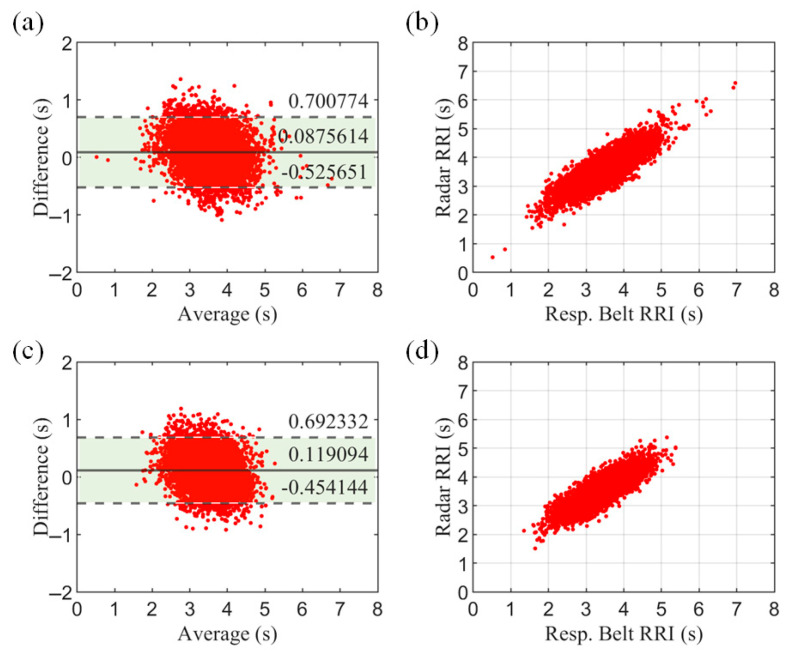
The results of statistical analysis of RRIs. (**a**) a Bland–Altman plot of chest measurements, with the upper and lower dashed lines indicating 95% limits of agreement, and the horizontal line in the middle indicating the mean of the difference between the radar and respiratory belt measurements. (**b**) a correlation scatterplot of radar versus respiratory belt measurements of chest data. (**c**) and (**d**) a Bland–Altman plot and correlation scatterplot of abdominal measurements, respectively.

To further evaluate the system, we assessed the respiratory cycle. The respiratory cycle—also known as the respiratory–respiratory interval (RRI)—provides more specific information than the respiratory rate (RR) and is a more refined respiratory characteristic. Figure 18 shows the Bland–Altman plot and correlation scatterplot for all subjects. The results show a high agreement between the radar measurements and the respiratory belt reference values.

To evaluate efficiency, we measured the end-to-end latency on a 2.5 GHz quad-core i5-7300HQ laptop. Processing 30 s of data (5340 samples) took 0.32 ± 0.08 s (n = 100 runs). The algorithm has low complexity and is readily portable to resource-constrained embedded platforms for real-time vital-sign monitoring.

### 5.2. Conditionality of System

To verify the generalization of the method, experiments were performed in different postures and the results are displayed in Table 4. The radar was fixed overhead using a stand about 50 cm from the body. Tests were conducted in supine, lateral, and prone postures and compared with the sitting posture. The results showed that there was no significant difference between the supine position and the sitting position. Measurement accuracy was severely reduced in the lateral position due to the reduced effective reflective area of the thoracic and abdominal cavities to the radar, as well as the weakening or loss of respiratory signals picked up by the radar due to limb occlusion. The measurement accuracy is somewhat reduced in the prone position, which may be due to the fact that respiration is indirectly detected through dorsal respiratory compensation, resulting in a low SNR. Especially in the abdominal position, it is difficult to observe undulations.

To validate the applicability of the system in real-world scenarios such as a hospital wards, we evaluated the effect of different coverings on the performance. The results are summarized in Table 5. Coverings include cotton garments, polyester garments, thin quilts, and thick blankets to cover common scenarios in the home or clinic. Subjects were placed in a supine position with the radar 50 cm away from the body.

As shown in Table 5, there is no significant difference in measurement performance when using cotton garments, polyester garments, or thin quilts as coverings. This suggests that the 120 GHz radar can effectively penetrate these materials without compromising measurement accuracy. However, in the case of thick blankets, the SNR of the IF signal is significantly reduced due to severe attenuation of the millimeter wave. As a result, the human target cannot be reliably identified in the range profile, making accurate measurement challenging.

### 5.3. Comparison with Related Work

Accurate and long-term monitoring of breathing patterns is a key task in health monitoring, especially in cases where traditional contact sensors cause user discomfort. Radar-based noncontact sensors provide a more convenient and comfortable solution. We have extensively compared our system with related work and summarized it in Table 6.

With the advancement of medical care and the increase in the number of patients with respiratory diseases, the observation of respiratory movements in the chest and abdomen is receiving more attention. Therefore, our system focuses on more respiratory parameters, such as respiratory frequency, depth, and TAA, aiming to achieve a more complete description of respiratory patterns to determine the health of the subjects.

In summary, our proposed dual radar system integrates advanced radar technology and customized signal processing algorithms to provide a comprehensive solution for noncontact respiratory monitoring. Compared to related works, our system offers enhanced accuracy, robustness against motion interferences, and the ability to measure TAA, making it a promising tool for both clinical and home-based health monitoring applications.

### 5.4. Limitation and Future Works

Our system still has some limitations. First, the current experiments are limited in sample size and diversity, which restricts the validation of our findings to a broader range of patients with various respiratory conditions. Second, the calculation of the error in the TAA phase angle used the respiratory band as the true value and did not compare the results with the gold standard of respiratory inductance plethysmography (RIP), in addition to a lack of validation of the sensitivity and specificity of tiered diagnosis in patients with COPD. Third, the parameters in the waveform reconstruction algorithm were derived from simulation experiments. Although good results have been achieved for data from healthy individuals and COPD patients, they may not apply to critically ill patients with complex breathing patterns. The thresholds for determining CSR versus Biot’s respiration in the pathological breathing pattern recognition method are empirical and need to be validated with more clinical data. Fourth, the validation of apnea detection was simulated through breath-holding experiments, which did not validate the ability to identify and differentiate between obstructive and central sleep apnea. Finally, the ease of use of the system needs to be improved to facilitate precise control of the chest and abdomen measurement positions.

To address the above issues, we plan to conduct the following studies in the future. First, we plan to expand our subject population for more comprehensive testing. We will conduct stratified analyses based on disease type and severity, body mass index (BMI), and age groups. Obese, elderly, and pediatric populations will also be included to validate the generalizability of the system. Second, the performance of the system for TAA phase angle calculation will be validated using the RIP gold standard, and a large-scale clinical study will be conducted on patients with COPD of varying severity to validate sensitivity and specificity. Third, we will further validate the adaptability of the filter parameters using test data from patients with different breathing patterns and study the adaptive parameter selection approach to improve the generalization of the system. Fourth, multimodal vital sign measurements will be performed in combination with other devices such as oro-nasal airflow sensors and blood oxygen sensors, to improve the accuracy of the system for disease diagnosis through different physiological parameters. Finally, we will design a controlled pointing device for easy use by clinicians.

## 6. Conclusions

This study presented a dual radar system for noncontact measurement of comprehensive breathing patterns using 120 GHz FMCW radars. The system enables independent monitoring of chest and abdominal movements through narrow-beam antennas and signal design. Key features include accurate respiratory waveform reconstruction via Savitzky–Golay filtering, automatic motion interference suppression, reliable pathological respiratory pattern recognition, and the detection of paradoxical breathing using cross-correlation. The system demonstrated a high correlation with reference respiratory belt signals (correlation coefficient: 0.92) and accurate respiratory rate estimation (RMSE: 0.80 bpm). Future work will focus on expanding the sample and diversity of the subject population to facilitate progress in its clinical application. Overall, the proposed system offers a reliable solution for noncontact respiratory monitoring, aiding in disease diagnosis and prolonged health monitoring.

## Figures and Tables

**Table 1:** Radar signal parameters.

Radar Parameter	Value
$\mathrm{Starting}\;\mathrm{Frequency}:\;f_{0}$	119 GHz
Bandwidth: B	6 GHz
$\mathrm{Ramp}\;\mathrm{Time}:\;T_{ramp}$	1.2 ms
$\mathrm{Idle}\;\mathrm{Time}:\;T_{idle}$	4.4 ms
Pulse Repetition Frequency: PRF	178 Hz
$\mathrm{ADC}\;\mathrm{Sampling}\;\mathrm{Frequency}\;\mathrm{of}\;\mathrm{IF}\;\mathrm{signal}:\;f_{s}$	250 kHz
$\mathrm{Dual}\;\mathrm{Radar}\;\mathrm{Delay}:\;T_{delay}$	2.8 ms

**Table 2:** The basic information and health status of subjects.

Subject	Gender	Age	BMI	Healthy Status	Subject	Gender	Age	BMI	Healthy Status
1	Male	23	22.5	Healthy	20	Female	24	23.6	Healthy
2	Male	25	23.1	Healthy	21	Female	33	30.1	Healthy
3	Male	30	27.8	Healthy	22	Female	29	28	Healthy
4	Male	28	29.2	Healthy	23	Female	23	22.7	Healthy
5	Male	22	21.9	Healthy	24	Female	27	25.1	Healthy
6	Male	26	24.3	Healthy	25	Female	21	21.8	Healthy
7	Male	24	23.7	Healthy	26	Female	24	23.2	Healthy
8	Male	29	26.5	Healthy	27	Female	28	26.7	Healthy
9	Male	27	25.8	Healthy	28	Female	25	24.5	Healthy
10	Male	21	22.1	Healthy	29	Female	31	28.9	Healthy
11	Male	31	28.3	Healthy	30	Female	22	22	Healthy
12	Male	24	23.4	Healthy	31	Female	46	25	Healthy
13	Male	42	29.5	Healthy	32	Female	24	23.5	Healthy
14	Male	23	22.8	Healthy	33	Female	30	27.9	Healthy
15	Male	30	27.6	Healthy	34	Male	23	22.6	Healthy
16	Male	23	24.1	Healthy	35	Male	27	25.3	Healthy
17	Male	58	26.2	Healthy	36	Male	48	20.3	COPD
18	Male	42	22.3	Healthy	37	Female	56	18.1	COPD
19	Male	26	25.4	Healthy	38	Male	68	18.2	Heart Failure

**Table 3:** The performance of radar signals and reference respiratory belt signals.

Subject	Chest		Abdomen		TAA		Subject	Chest		Abdomen		TAA	
	Corr.	RMSE	Corr.	RMSE	SD	MAE		Corr.	RMSE	Corr.	RMSE	SD	MAE
1	0.9641	0.63	0.9233	0.86	4.1°	3.3°	20	0.9819	0.57	0.9818	0.84	2.8°	2.5°
2	0.9374	0.73	0.9829	0.49	3.2°	2.6°	21	0.8670	0.93	0.8752	0.95	3.2°	3.9°
3	0.8813	0.91	0.8345	1.08	4.5°	4.7°	22	0.8283	1.05	0.9886	0.88	3.7°	3.6°
4	0.8455	1.01	0.8497	0.89	7.2°	5.8°	23	0.9655	0.79	0.9464	0.52	3.6°	2.9°
5	0.9647	0.54	0.9384	0.71	3.5°	2.8°	24	0.9454	0.90	0.9224	0.76	3.5°	3.2°
6	0.9434	0.66	0.9143	0.85	6.2°	3.8°	25	0.9743	0.42	0.9820	0.39	4.7°	3.1°
7	0.9354	0.81	0.9456	0.78	4.0°	3.3°	26	0.9412	0.65	0.9487	0.62	4.0°	3.1°
8	0.9102	0.82	0.9256	0.79	4.4°	3.2°	27	0.9001	0.89	0.9089	0.86	4.4°	3.5°
9	0.8945	0.88	0.9012	0.85	4.0°	3.7°	28	0.9245	0.74	0.9301	0.72	3.0°	3.2°
10	0.9321	0.71	0.9401	0.68	3.5°	2.9°	29	0.8832	0.97	0.889	0.94	5.5°	4.2°
11	0.8876	0.95	0.8923	0.91	6.3°	4.1°	30	0.9367	0.68	0.9423	0.66	3.2°	2.9°
12	0.9188	0.78	0.9234	0.76	4.5°	3.0°	31	0.9012	0.91	0.9078	0.88	4.5°	3.6°
13	0.8754	1.02	0.8812	0.98	5.6°	4.5°	32	0.9289	0.73	0.9345	0.70	3.7°	3.0°
14	0.9201	0.76	0.9256	0.74	5.0°	3.2°	33	0.8798	1.02	0.8856	0.97	5.4°	4.3°
15	0.8823	0.98	0.8881	0.95	3.8°	4.0°	34	0.9134	0.69	0.919	0.77	3.6°	2.9°
16	0.9156	0.80	0.9201	0.78	4.3°	3.3°	35	0.9067	0.87	0.9123	0.84	4.3°	3.5°
17	0.889	0.93	0.8945	0.90	3.8°	3.9°	36	0.9773	0.76	0.9646	0.75	3.7°	2.9°
18	0.9389	0.67	0.9445	0.65	3.7°	2.9°	37	0.9011	1.09	0.9331	0.81	4.3°	3.1°
19	0.9023	0.89	0.9078	0.86	4.5°	3.6°	38	N/A	N/A	0.9126	0.63	N/A	N/A
Mean	0.92	0.81	0.92	0.79	4.3°	3.4°							

The heart failure patient could not tolerate the chest belt, so only abdominal data were available.

**Table 4:** A comparison of performance under different postural conditions.

Posture	Chest		Abdomen		TAA	
	Corr.	RMSE	Corr.	RMSE	SD	MAE
Sitting	0.9641	0.63	0.9233	0.86	4.1°	3.3°
Supine	0.9584	0.65	0.9365	0.78	4.3°	3.4°
Lateral	0.2145	15.79	0.1754	18.65	202.8°	156.1°
Prone	0.7241	3.83	0.4384	11.21	62.5°	56.3°

**Table 5:** A comparison of performance under different coverings conditions.

Covering	Chest		Abdomen		TAA	
	Corr.	RMSE	Corr.	RMSE	SD	MAE
Cotton garments	0.9584	0.65	0.9365	0.78	4.1°	3.4°
Polyester garments	0.9512	0.64	0.9184	0.88	3.9°	3.3°
Thin quilts	0.9217	0.71	0.9111	0.89	4.2°	3.4°
Thick blankets	N/A	N/A	N/A	N/A	N/A	N/A

Under the covering of thick blankets, the radar signal was severely attenuated and the SNR was too low to effectively extract the respiratory signal, so there were no valid measurement data.

**Table 6:** A comparison of our system with related work.

Ref	Year	Radar System	Results	Motion Suppression	TAA Detection
[44]	2024	60 GHz FMCW Triple Radar System	RR: ±3 BPM Agreement: 98.5% ±1 BPM Agreement: 93.2%	No	No
[45]	2024	60 GHz FMCW Dual Radar System	RR: Max error is 1.9 bpm	Yes	No
[46]	2025	77 GHz FMCW Single Radar System	TAA: Mean error is 1.56°, MAE is 4.01°	No	Yes
Ours	2025	120 GHz FMCW Dual Radar System	RR: RMSE is 0.80 bpm TAA: MAE is 3.4°	Yes	Yes

## Data Availability

The data presented in this study are available on request from the corresponding author.
